# Spherules and IBV

**DOI:** 10.4161/bioe.29323

**Published:** 2014-06-04

**Authors:** Helena J Maier, Philippa C Hawes, Sarah M Keep, Paul Britton

**Affiliations:** 1 The Pirbright Institute; Compton Laboratory; Compton, UK; 2 The Pirbright Institute; Pirbright Laboratory; Pirbright, UK

**Keywords:** coronavirus, infectious bronchitis virus, membrane rearrangements, double membrane vesicles, spherules

## Abstract

Infectious bronchitis virus (IBV) is an economically important virus infecting chickens, causing large losses to the poultry industry globally. While vaccines are available, there is a requirement for novel vaccine strategies due to high strain variation and poor cross-protection. This requires a more detailed understanding of virus-host cell interactions to identify candidates for targeted virus attenuation. One key area of research in the positive sense RNA virus field, due to its central role in virus replication, is the induction of cellular membrane rearrangements by this class of viruses for the assembly of virus replication complexes. In our recent work, we identified the structures induced by IBV during infection of cultured cells, as well as primary cells and ex vivo organ culture. We identified structures novel to the coronavirus family, which strongly resemble replication sites of other positive sense RNA viruses. We have begun to extend this work using recombinant IBVs, which are chimera of different virus strains to study the role of viral proteins in the induction of membrane rearrangements.

## Introduction

Infectious bronchitis virus (IBV) is a gammacoronavirus that infects poultry primarily, causing an infectious respiratory disease. In addition, infection results in substantial economic losses to the worldwide poultry industry as a result of reduced egg quality, egg production, and meat quality. Although vaccines are available, there is a large degree of variation between strains of IBV and poor cross-protection. As a result, novel strategies are required to develop more effective vaccines. To enable this, it is important to understand the interaction between the virus and the host cell, allowing future manipulation and targeted attenuation of the virus by reverse genetics to develop vaccine strains. A critical step in the replication of all positive sense single stranded RNA (+RNA) viruses is the induction of cellular membrane rearrangements, providing the virus with a platform for the assembly of replication complexes responsible for synthesizing viral RNA. It can be considered that a site for viral RNA synthesis would provide an enclosed environment that protects viral RNA from cellular detection, preventing host degradation of RNA and preventing cellular immune stimulation. However, the site must also allow for the exchange of material with the cytoplasm. Nucleotides and other cellular reagents must enter to allow RNA synthesis to occur and viral RNA must exit for translation and assembly of new virus particles. In broad terms, the type of membrane rearrangements induced by different +RNA viruses can be split into two groups: double membrane vesicles (DMVs) and spherules.^1^ Viruses that induce DMV type structures include poliovirus (PV) and hepatitis C virus (HCV). In PV infected cells, single membrane structures are induced early in infection, followed by double membrane structures.^2^ They seem to be derived from the cellular ER and viral replicase proteins have been found to localize to the cytoplasmic side of both single and double membrane structures.^2^^,^^3^ In the case of HCV, DMVs are derived from the endoplasmic reticulum (ER) and at early time points, the outer membrane remains attached to the ER. By later time points, the DMVs appear to bud and become free vesicles. The majority of DMVs were found to be sealed from the cytoplasm with only 10% having a visible pore from the interior.^4^ Viruses that induce spherule type structures, or invaginated vesicles, include Semliki Forest virus (SFV), Flock House virus (FHV), Brome Mosaic virus (BMV), and the *Flaviviruses* Dengue virus (DENV) and West Nile virus (WNV). For all of these viruses, the spherule-like structure is composed of an invagination of a single membrane derived from a cellular structure. SFV induces spherules at the plasma membrane early in infection, which later become internalized as part of the endo/lysosomal pathway.^5^ FHV induces spherules on the outer mitochondrial membrane^6^ and BMV, DENV, and WNV all induce spherules or invaginated vesicles on the ER.^7^^-^^10^ For each virus, replicase proteins and RNA have been found to localize to spherules^5^^,^^8^^,^^9^^,^^11^ and there is an 8–10 nm pore connecting the spherule interior with the cytoplasm.^6^^,^^9^^,^^10^^,^^12^

It has long been characterized that in continuous cell culture, coronaviruses induce the formation of double membrane vesicles (DMVs) and branching networks of membranes known as convoluted membranes.^13^^-^^21^ These structures have been identified in cells infected with three different betacoronaviruses: mouse hepatitis virus (MHV), severe acute respiratory syndrome coronavirus (SARS-CoV), and the recently identified Middle East respiratory syndrome (MERS)-CoV. DMVs provide an enclosed environment and have historically been proposed as the site of assembly of viral replication complexes. Some viral replicase proteins have been shown to be located inside DMVs or on their membranes.^14^^-^^19^ In addition, virus associated dsRNA, a potential replicative intermediate, is located on the interior of DMVs.^18^ In more recent work utilizing electron tomography allowing 3D reconstruction of membrane structures, it was demonstrated that in SARS-CoV infected continuous cell culture, these structures are derived from and remain connected to the cellular ER.^18^ In addition, using this technique Knoops et. al. were able to study in detail the membrane continuity of these vesicles. Interestingly, they were unable to find any pores or connections between the interior of the DMV and the cell cytoplasm.^18^ This raised the question, if DMVs are the site of RNA synthesis, how does newly synthesized RNA exit the compartment?

Other work has subsequently questioned of the role of dsRNA during coronavirus replication. Although at early time points post-infection dsRNA was found to co-localize with nascent RNA, at later time points this co-localization did not occur.^22^ This suggests that dsRNA cannot always be presumed to provide a marker for sites of active RNA synthesis and DMVs may provide a site to shield non-productive RNA from cellular detection, rather than provide a site for viral RNA synthesis.

## Membrane Structures in IBV Infected Cells

Although first discovered in 1933, in recent years the molecular characterization of the interaction between avian gammacoronavirus IBV and the host cell has lagged behind work on the betacoronaviruses MHV, SARS-CoV, and MERS-CoV. However, an advantage of working with a poultry pathogen is the ability to perform studies in primary cells of the native host, as well as ex vivo organ culture. This provides critical validation that the observations made are relevant to the natural infection setting in a whole animal and are not a result of use of transformed cell lines. In our recent work we characterized the membrane rearrangements induced by IBV during infection of mammalian Vero cells, primary chick kidney cells, and ex vivo tracheal organ culture. In all three systems, we found that IBV induced novel membrane structures not found in previous work studying betacoronaviruses. IBV infection induced regions of the ER to become zippered together forming closely paired membranes and small double membrane invaginations, or spherules, which were tethered to the zippered ER.^23^ Significantly, unlike any previously identified coronavirus induced structure, use of electron tomography demonstrated that spherules contain a 4.4 nm channel or pore connecting their interior to the cytoplasm of the cell.^23^ The availability of improved imaging techniques, like electron tomography, was invaluable to this work. The ability to reconstruct and visualize membranes in three dimensions allowed a detailed study of connections between different structures to be performed. Using standard electron microscopy techniques, examples of tethered spherules were in the minority, with most appearing as discrete vesicles in the cytoplasm. However, electron tomography provided evidence that the vast majority of spherules, if not all, are tethered to the zippered ER and provided evidence of a pore connecting the spherule to the cytoplasm.

In addition to the novel structures described above, IBV was also observed to induce DMVs very similar in structure to those previously identified in cells infected with SARS-CoV, MHV, and MERS-CoV. The similarity of the structure of DMVs induced by IBV and the previously studied coronaviruses suggests that these structures perform similar functions during replication of all coronaviruses. However, zippered ER and spherules show a marked structural difference to convoluted membranes observed during replication of the betacoronaviruses. Convoluted membranes showed little by way of organized structure when visualized by electron tomography,^18^ whereas zippered ER was visualized as a regular track of paired membranes surrounded by an electron dense region. In addition, spherules tethered to the zippered ER were of a uniform size and appearance. Most interestingly, these structures exhibit a strong similarity to spherules induced by other families of +RNA viruses. For these viruses, spherules have been shown to be the site of viral RNA synthesis and are induced by viral replicase proteins, sometimes requiring an interaction with viral RNA.^5^^,^^8^^,^^24^ One interesting difference between spherules induced by IBV and other positive sense RNA viruses is that IBV induces spherules composed of a double membrane instead of a single membrane. In addition, other viruses form spherules on membranes of existing cellular structures, i.e., mitochondria or the ER. IBV, however, appears to modify a cellular membrane prior to the induction of spherule formation. The reason for this is unknown, although it is possible that this may reflect the increased number of viral proteins that comprise the replication complex of coronaviruses compared with other +RNA viruses.

The potential location of replication complexes on the different membrane structures was also considered. The role of dsRNA during virus replication remains undetermined, but, similar to other coronaviruses, it is likely to be contained within DMVs in IBV infected cells. However, viral RNA dependent RNA polymerase, nsp12, must be present at the site of RNA synthesis so was used as an alternate marker for replication complexes. Accumulation of dsRNA in infected primary cells can be detected by immunofluorescence labeling from 4 hpi so co-localization of dsRNA and nsp12 was compared at 4, 6, and 8 hpi. At all of these time points, less than 1.5% of total dsRNA or nsp12 signal was co-localized. If we assume that dsRNA is located on the interior of DMVs, this data suggests that nsp12 and the site of active RNA synthesis cannot be located within DMVs.

Taking our observations as a whole, we hypothesize that in IBV infected cells, (1) DMVs function to shield excess or non-productive virus induced dsRNA from the cell and (2) viral replication complexes assemble on the interior of spherules tethered to the zippered ER (Fig. 1). DMVs would function to prevent the stimulation of the cellular interferon response, aiding virus replication. As coronaviruses are released from infected cells by exocytosis rather than in a single lytic event, progeny continue to assemble and be released from cells for extended periods of time. Therefore, strategies will be required to control cellular responses to virus infection for long periods. Spherules, on the other hand, provide an archetypal replication site concentrating viral enzymes and protecting viral RNA yet, significantly, allowing exchange of material with the cytoplasm.

**Figure:**
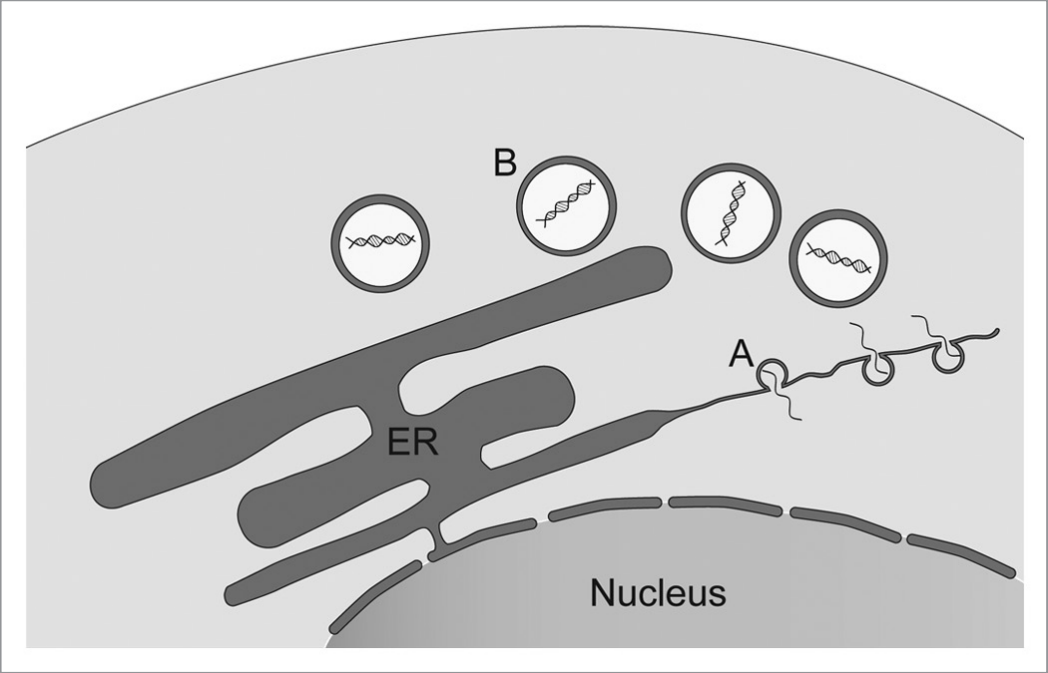
**Figure 1.** Model of function of membrane structures induced during IBV infection. During replication, IBV induces the formation of regions of zippered ER with associated spherules (**A**), allowing viral replication-transcription complexes (RTCs) to form and viral RNA synthesis to occur. As a by-product of viral RNA synthesis, non-productive dsRNA is produced and shielded from cellular detection inside DMVs (**B**).

Following on from this work, it will be interesting to identify the viral proteins responsible for controlling the rearrangement of cellular membranes. Disruption or alteration of the function of these proteins by reverse genetics may provide good candidates for virus attenuation for vaccine production. Coronaviruses express three membrane bound non-structural proteins (nsps): nsp3, nsp4, and nsp6. By analogy with previous work studying distantly related arteriviruses,^25^^,^^26^ these membrane proteins are the most likely candidates. Indeed, in two recent studies where these proteins from SARS-CoV and MHV have been expressed in cells outside the context of whole virus have suggested that this is the case.^27^^,^^28^ The availability of recombinant viruses which are chimera of two different IBV strains also provides an opportunity to examine the role of viral proteins in the induction of membrane rearrangements. We examined the ability of two different chimeric viruses to induce membrane rearrangements. The first virus, rBeauR-Rep-M41-Struct, contains the replicase polyprotein gene expressing the nsps from apathogenic Beaudette and the structural and accessory genes from pathogenic M41.^29^ The second virus, rBeauR-nsp2-14-M41-nsp15-Struct, contains the nsp2-nsp14 region of the polyprotein gene from Beaudette and nsp15-nsp16 plus the structural and accessory genes from pathogenic M41. When analyzed by transmission electron microscopy, both viruses were found to induce membrane structures analogous to those observed in cells infected with wild type virus used in our previous work, with zippered ER, spherules, and DMVs all present (Fig. 2). Extension of this work using viruses with strain mismatches for the three membrane bound non-structural proteins, along with ectopic expression of these nsps, will be valuable in the future to further analyze the role of these proteins in membrane rearrangements.

**Figure:**
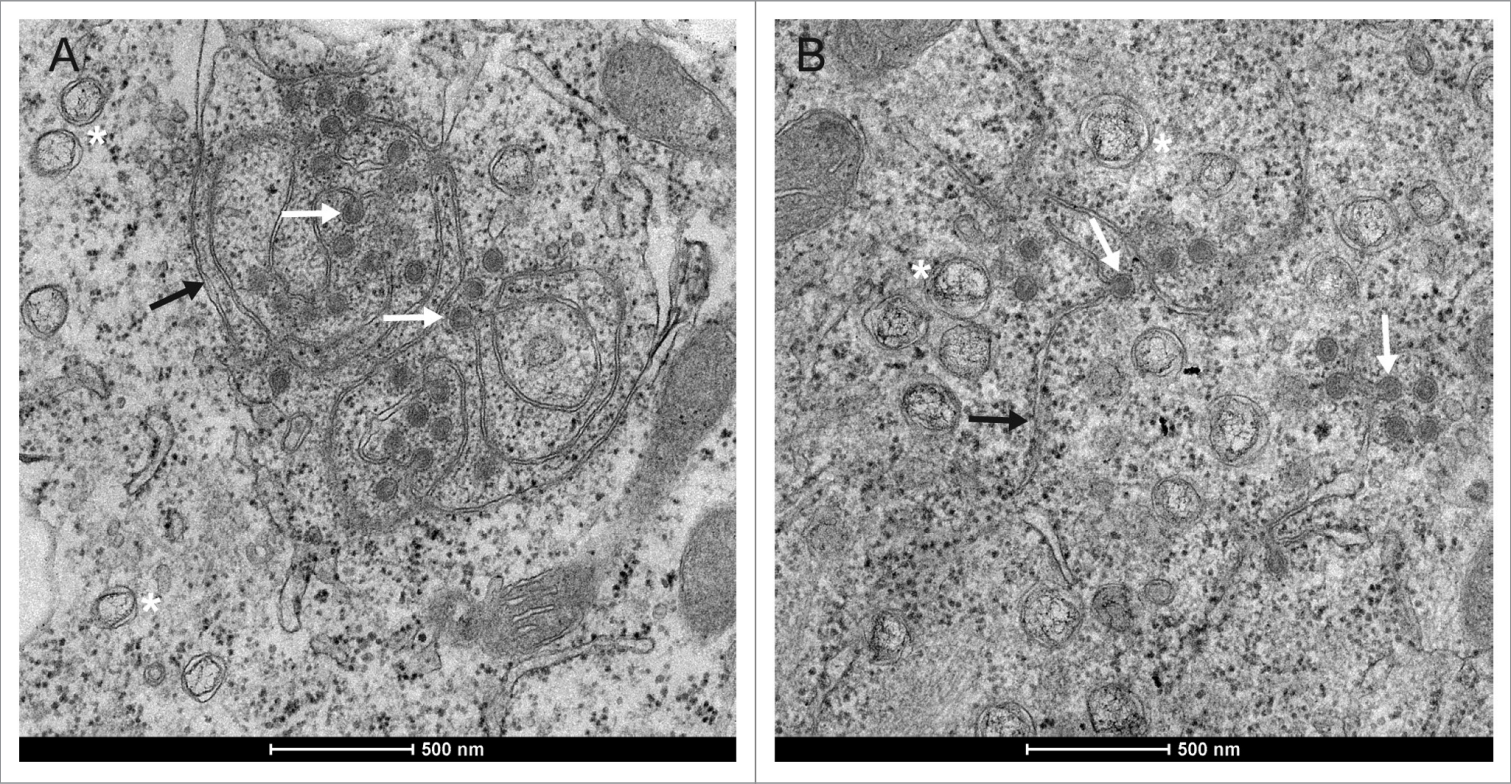
**Figure 2.** Zippered ER, spherules and double membrane vesicles induced by chimeric IBVs. Tracheal organ cultures were infected with rBeauR-Rep-M41-Struct (**A**) or rBeauR-nsp2-14-M41-nsp15-Struct (**B**) and chemically fixed at 24 h post infection. Zippered ER (black arrows), spherules (white arrows), and DMVs (asterisks) could be detected. Scale bars indicate 500 nm.

## Abbreviations:

DMVs: double membrane vesicles
IBV: infectious bronchitis virus
+RNA: positive sense RNA virus

## Notes

10.4161/bioe.29323

MaierHJHawesPCCottamEMMantellJVerkadePMonaghanPWilemanTBrittonP Infectious bronchitis virus generates spherules from zippered endoplasmic reticulum membranes MBio 2013 4 e00801 13 10.1128/mBio.00801-13
